# Do beta-blockers reduce negative intrusive thoughts and anxiety in cancer survivors? – An emulated trial

**DOI:** 10.1186/s12885-024-12236-3

**Published:** 2024-04-11

**Authors:** Carolina Ehrencrona, Ying Li, Eva Angenete, Eva Haglind, Stefan Franzén, Anna Grimby-Ekman, David Bock

**Affiliations:** 1 Department of Surgery, SSORG - Scandinavian Surgical Outcomes Research Group, Institute of Clinical Sciences, Sahlgrenska Academy, University of Gothenburg, Gothenburg, Sweden; 2 Department of Surgery, Region Västra Götaland, Sahlgrenska University Hospital, Gothenburg, Sweden; 3 Medical & Payer Evidence Statistics, BioPharmaceuticals R&D, AstraZeneca, Gothenburg, Sweden; 4 Biostatistics, School of Public Health and Community Medicine, Institute of Medicine, Sahlgrenska Academy, University of Gothenburg, Gothenburg, Sweden; 5 Early Biometrics and Statistical Innovation, Data Science & AI, BioPharmaceuticals R&D, AstraZeneca, Gothenburg, Sweden

**Keywords:** Adrenergic beta-antagonists, Cancer survivors, Colorectal cancer, Trial emulation, Intrusive thoughts, Prostate cancer, Psychological Distress, Quality of life

## Abstract

**Background:**

High rates of negative intrusive thoughts have been reported among cancer patients. Prevalent users of beta-blocker therapy have reported lower levels of cancer related intrusive thoughts than non-user. The aim of this study is to investigate if initiation of beta-blocker therapy reduces the prevalence and severity of intrusive thoughts (co-primary endpoints) and the prevalence of anxiety, depressed mood, and low quality of life (secondary endpoints) in cancer survivors.

**Methods:**

Data on patient-reported outcomes from three cohort studies of Swedish patients diagnosed with colon, prostate or rectal cancer were combined with data on beta-blocker prescriptions retrieved from the Swedish Prescribed Drug Register. Two randomized controlled trials were emulated. Trial 1 had follow-up 1 year after diagnosis, trial 2 had follow-up 2 years after diagnosis, baseline in both trials was 12 months before follow-up. Those who initiated beta-blocker therapy between baseline and follow-up was assigned Active group, those who did not was assigned Control group. All endpoints were analysed using Bayesian ordered logistic regression.

**Results:**

Trial 1 consisted of Active group, *n* = 59, and Control group, *n* = 3936. Trial 2 consisted of Active group, *n* = 87, and Control group, *n* = 3132. The majority of participants were men, 83% in trial 1 and 94% in trial 2. The prevalence and severity of intrusive thoughts were lower in the Active group in trial 1, but no significant differences between groups were found in either trial. The prevalence of depressed mood, worse quality of life and periods of anxiety were higher in the Active group in both trials with significant differences for quality of life in trial 1 and anxiety in trial 2.

**Conclusions:**

The emulated trials demonstrated no evidence of a protective effect of beta-blocker therapy against intrusive thoughts. The Active group had reduced quality of life and elevated anxiety compared to the Control group.

**Trial registration:**

The three cohort studies were registered at isrctn.com/clinicaltrials.gov (ISRCTN06393679, NCT02530593 and NCT01477229).

**Supplementary Information:**

The online version contains supplementary material available at 10.1186/s12885-024-12236-3.

## Background

The patient’s journey after receiving a cancer diagnosis can be a highly distressing experience with several stressful and traumatic events. Stressors that cause psychological distress and intrusive thoughts include uncertainties surrounding cancer treatment and prognosis, and the threat posed on the individual’s life and normal way of living [1]. Post-traumatic stress disorder (PSTD) is more common in cancer survivors than controls without a history of cancer [2]. One symptom of PTSD is intrusive thoughts of past events, which are defined as unwanted, unintended, recurrent thoughts causing distress [3]. High rates of intrusive symptoms have been reported by patients with cancer, including thoughts of past events and future threats the cancer poses [4]. Intrusive thoughts have also been linked to anxiety as well as depression [5] and have been identified as a predictor for depressed mood, waking up with anxiety and lower quality of life up to two years after prostate cancer surgery [6]. For rectal cancer patients intrusive thoughts are associated with lower quality of life both at diagnosis [7] and 3 years after surgery [8].

Beta-blockers, typically used for the management of cardiovascular diseases, have been explored as pharmacological treatment to PTSD and anxiety. Propranolol, given closely after, or, before and after the reactivation of a traumatic memory, can reduce both the intensity and the frequency of these memories as well as emotional responses with long-lasting effects [9]. Prevalent users of beta-blockers show lower levels of anxiety-related distress than non-users after the death of a person close to them [10]. Moreover, in a cross-sectional single-center study in patients with colorectal and breast cancer, current users of beta-blockers reported lower levels of intrusive thoughts than non-users [11].

This study combined three longitudinal cancer cohort studies with registry data of pharmacological prescription and dispensation to explore if beta-blocker treatment can reduce intrusive thoughts in cancer survivors. To further strengthen the level of evidence and reduce the risk of bias when using observational data, we conducted two emulated target trials based on the framework presented by Hernán et al. [12]. These emulated trials aimed to assess whether prescribed beta-blockers were associated with reduced intrusive thoughts, anxiety, depressed mode, and higher quality of life among colorectal and prostate cancer survivors.

## Methods

### Participants and data sources

This target trial emulation used data from three prospective longitudinal multicenter cohort studies of patients diagnosed with prostate cancer (*n* = 3705) recruited between 2008–2011, rectal cancer (*n* = 1215) recruited between 2012–2015, and colon cancer (*n* = 1891) recruited between 2015–2020. The three studies were registered at isrctn.com/clinicaltrials.gov (ISRCTN06393679, NCT02530593 and NCT01477229). Ethical approvals were obtained from the Regional Ethical Review Boards in Göteborg, Sweden for LAPPRO (Laparoscopic Prostatectomy Robot Open), (EPN 277–07), for QoLiRECT (Quality of Life in Rectal Cancer) in Göteborg, Sweden (EPN 595–11) and Denmark (H-3–2012-FSP26), and for QoLiCOL (Quality of Life in Colon Cancer) in Göteborg, Sweden (EPN 957–14) and Denmark (H-16027323). All patients gave informed consent to participate in the studies. LAPPRO was primarily designed for comparing robot-assisted laparoscopic with open retropubic radical prostatectomy. QoLiRECT and QoLiCOL was designed for exploring patient-reported outcomes after treatment for rectal or colon cancer.

Data on prescriptions of beta-blockers (Anatomic Therapeutic Chemical classification, ATC, code C07) were retrieved from the Swedish Prescribed Drug Register (Läkemedelsregistret) for the period 1 year before and up to 2 years after study inclusion. Information on prescriptions of anti-depressants (ATC code N06A) was also retrieved from the registry. Since we could only retrieve prescription data for Swedish patients the Danish patients in the cohorts were excluded. All three studies collected patient-reported data using a comprehensive questionnaire that was developed from themes identified during patient interviews and subsequently validated by experts and survivors of the respective cancer type. The creation and validation processes for the questionnaires have been previously described in detail [13, 14]. These questionnaires were distributed preoperatively and at 12 months to all patients and at 24 months for prostate and rectal cancer patients. There was no assessment at 24 months in the colon cancer study.

### Study design

Observational data was used to emulate hypothetical randomized target trials (Additional file 1, Table S1.1). The eligibility criteria of the target analysis were patients diagnosed with prostate, colon, or rectal cancer who had no previous use of beta-blockers. Those who fulfilled eligibility criteria and had an assessment of outcomes (i.e., completed questionnaires) were assigned to one of two strategies.

- Strategy 1: Initiate beta-blocker therapy between baseline and follow-up (Active group).
- Strategy 2: Refrain from taking beta-blocker therapy between baseline and follow-up (Control group).

The estimands of interest were the ratios (Active vs Control) of odds for poor outcome 12 months after randomization for the endpoints in Table 1, adjusted for baseline values using an intention-to-treat analysis. When estimating the statistical models there is a risk of convergence issues when some response categories of the endpoints have low observed frequencies. To avoid this, categories with very low prevalence were combined prior to statistical analysis.

**Table 1:** Outcome measures of primary and secondary endpoints

Endpoints	Questions	Categories in questionnaires	Categories used in analysis
Primary	How often during the past month have you had negative thoughts about your prostate/colon/rectal cancer, suddenly and unintentionally?	1 ‘Never’, 2 ‘More seldom than once a week’, 3 ‘At least once a week’, 4 ‘At least three times a week’, 5 ‘At least once a day’, 6 ‘At least three times a day’ and 7 ‘At least seven times a day’	1, 2, 3, 4–7 (combined)
Co-primary	How intrusive have you experienced the sudden negative thoughts about your prostate/colon/rectal cancer?	1. ‘Not applicable, I have not had any sudden thoughts about cancer’, 2. ‘Not at all intrusive’, 3. ‘A little bit intrusive’, 4. ‘Moderately intrusive’, 5. ‘Very intrusive’	1, 2, 3, 4–5 (combined)
Secondary 1	Would you call yourself depressed?	1. ‘No’, 2. ‘Yes’, 3. ‘I don’t know’	1, 2–3 (combined)
Secondary 2	How would you describe your quality of life during the past month?	0 (‘No quality of life’), 1, 2, 3, 4, 5, 6 (‘Best possible quality of life’)	0–3 (combined), 4, 5, 6
Secondary 3	Have you experienced period of intense anxiety, worry or panic in the last month (for example palpitations, shortness of breath or dizziness)?^a^	1 ‘Never’, 2 ‘More seldom than once a week’, 3 ‘At least once a week’, 4 ‘At least three times a week’, 5 ‘At least once a day’, 6 ‘At least three times a day’ and 7 ‘At least seven times a day’	1, 2, 3–7 (combined)

^a^In the colorectal cancer questionnaires, the question was phrased ‘Have you experienced worry or anxiety the last month?’ with a 7-step scale from ‘never’ to ‘all the time’

The causal path between initiation of beta-blockers and the outcomes were considered to be confounded by age, self-reported hazardous alcohol consumption, and poor mental health at baseline [15]. To adjust for these variables in the statistical analysis, we emulated a true randomization to the two strategies. Hazardous alcohol consumption was measured by the question “Have you had six glasses or more on the same occasion during the past month?” and categorized as “Yes” if this had happened on at least one occasion. Poor mental health was included as a covariate for all endpoints except for *Secondary 1 Depressed mood* because the baseline value of this endpoint is highly correlated with mental health. Poor mental health was defined as fulfilling at least one of three criteria: 1) retrieval of a prescription of anti-antidepressants during 12 months before baseline, 2) self-reported as seeking health care due to depression or psychiatric issues, or 3) answered “Yes” or “Don´t know” on the question: “Would you consider yourself depressed?”.

**Fig. 1:**
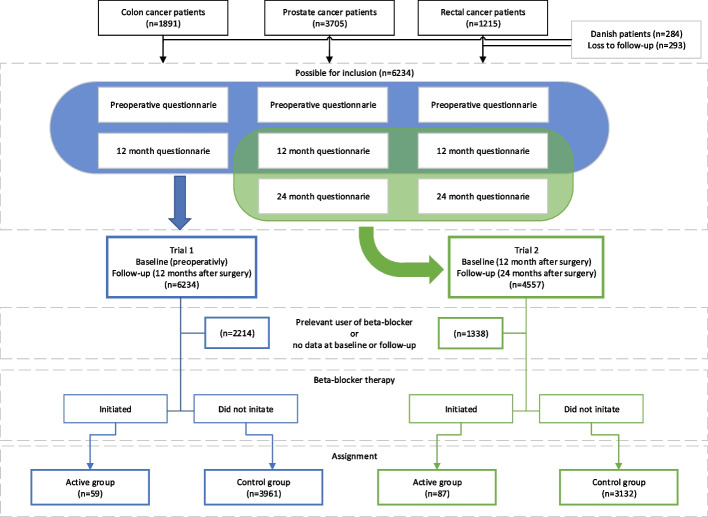
Flow chart of eligible and assigned patients in the emulated trials

Two trial emulations were performed. In trial 1, the preoperative questionnaire from the cohort studies was used as baseline, and the assessment 12 months after surgery as follow-up. In trial 2, the 12-month questionnaire was used as baseline, and at the assessment at 24 months after surgery as follow-up (Fig. 1). Eligible patients were those who had returned questionnaires to the study secretariat and had no dispensation of beta-blockers in the 12-month period before completing the baseline questionnaire. Given that patients in emulated trials do not necessarily initiate the treatment of interest at time zero (i.e., beta-blockers at baseline), there is a need to incorporate a grace period during which treatment initiation can occur [16]. In this study, the grace period was the entire period of 12 months between baseline and follow-up, during which at least one dispensation of beta-blockers (Active group) or no dispensation at all (Control group) occurred.

### Statistical analyses

All endpoints were analysed using a Bayesian ordered logistic regression model with proportional odds [17]. The confounding variables as well as baseline measurements of the respective endpoints were adjusted for by including them as covariates or factors in the analysis, where age was standardized (zero mean and unit variance). The baseline measurement of each endpoint was included in the model as an ordered categorical factor [18]. For the ordered categorical predictor, a Dirichlet prior was used. In the Statistical Analysis Plan (SAP), improper flat priors were prespecified for the intercept and the effects of treatment (Active vs Control) and the adjustment variables of age, binge drinking and signs of poor mental health. However, data sparsity in terms of the low number of patients assigned to the Active group gave rise to convergence issues. Therefore, weakly regularizing priors (Gaussian with zero mean and unit variance on the logit scale) were used in the analysis to ensure convergence of the Markov Chain Monte Carlo (MCMC) samplers. Missing data for endpoints and confounders were handled by multiple imputations (five imputations) using predictive mean matching [19]. Posterior draws were generated for each of the five sub-models using Hamiltonian Monte Carlo Sampling and were subsequently pooled to obtain the final posterior distributions. Results were presented as the posterior means and two-sided 95% credible intervals (CrI) for the odds ratio (OR) from the Bayesian ordered logistic regression model. Higher values (> 1) for the OR mean that patients in the Active group are worse off compared to those in the Control group. Lower values (< 1) mean that patients in the Active group are better off compared to those in the Control group. The posterior predictive distribution across the response categories was also visualized in graphs for each endpoint.

As sensitivity analyses, we performed unadjusted complete case analyses as well as adjusted frequentist analyses where the estimates from the five sub-models were pooled using Rubin’s rules (p. 76) [20]. In addition, analyses were performed of prevalent users of beta-blockers, with dispensation during the 12 months before diagnosis until follow-up. As this analysis does not have a defined time zero and consequently no defined baseline, the analyses were unadjusted. Additional details on the statistical analyses and programming code, results of the sensitivity analyses, convergence diagnostics of the MCMC samplers and characterization of the missing data are presented in the Additional file 1. All data analyses were made in R [21] using the brms [22], mice and MASS [23] packages.

## Result

Data from a total of 6234 patients were retrieved from the Swedish Prescribed Drug Register. Of these, 4020 patients were eligible for trial 1 and 3219 for trial 2 (Fig. 1). A total of 59 (trial 1) and 87 (trial 2) patients, who picked up at least one prescription of beta-blockers between baseline and follow-up, were assigned to the Active group.

**Fig. 2:**
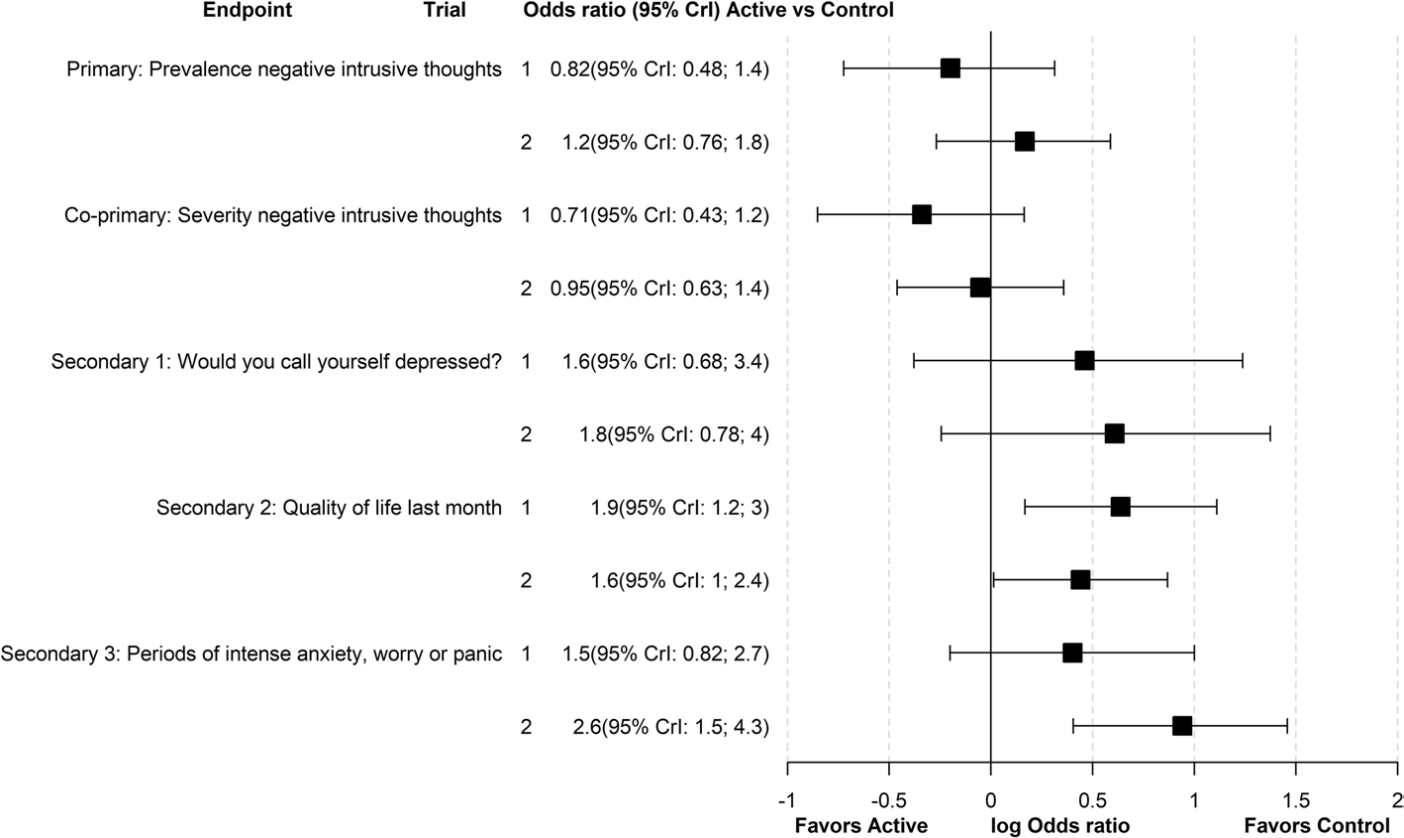
Forest plot of all endpoints. Analysis results posterior mean and 95% credible intervals (CrI) for the odds ratio (OR) for all endpoints

The majority of included patients had prostate cancer (64% trial 1, 86% trial 2) and were thus men (83% trial 1, 94% trial 2). Median age at baseline was 65 years (IQR 60, 70) in trial 1 and 64 years (IQR 59, 68) in trial 2. Demographic and disease characteristics are presented in Additional file 1, Table S1.2. Figure 2 present all endpoints with posterior mean and 95% credible intervals for the odds ratio.

**Fig. 3:**
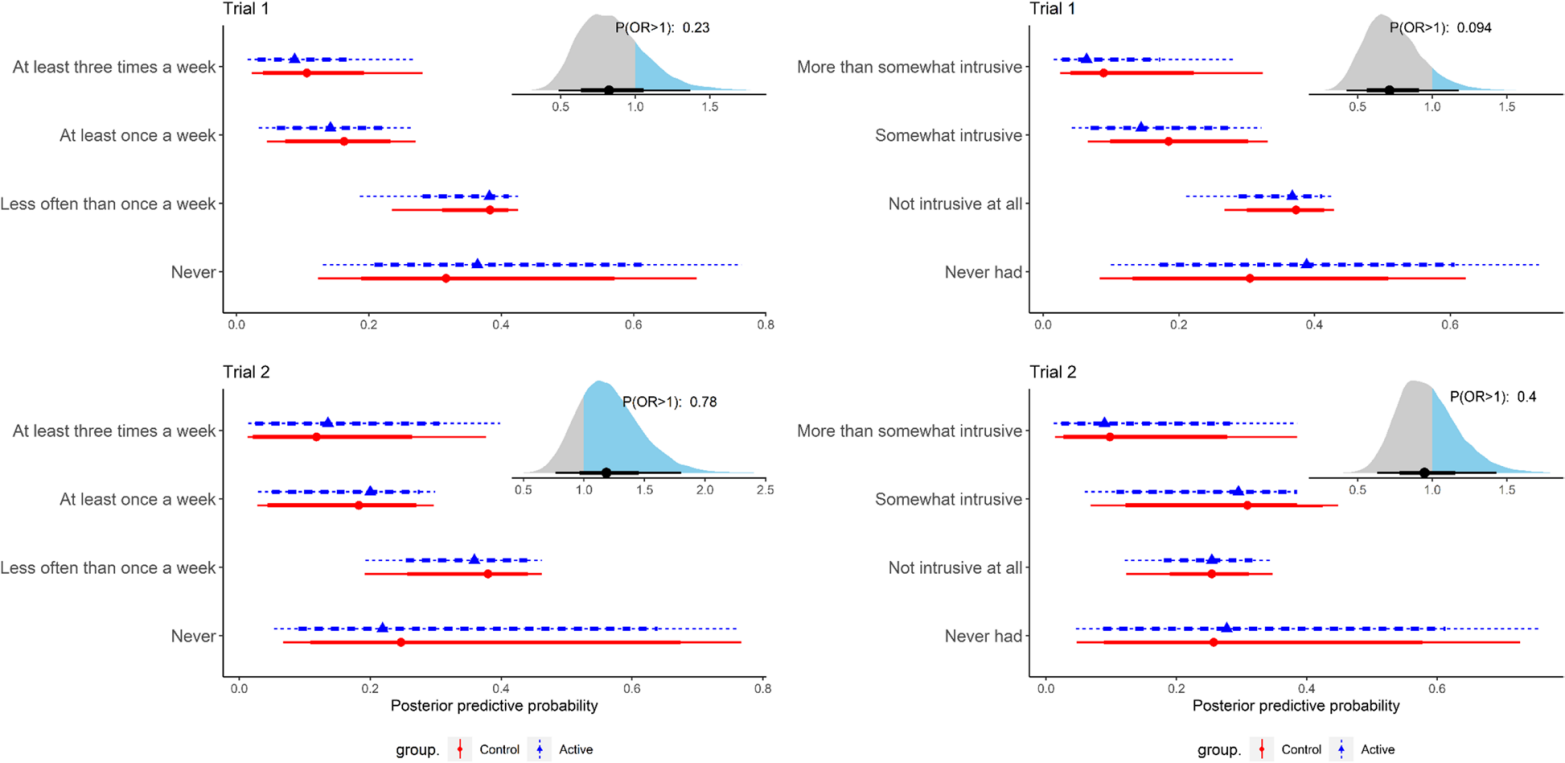
Negative intrusive thoughts about cancer. Posterior predictive distribution for the response categories and posterior distribution for the OR. Left panel: Prevalence. Right panel: Severity

For the co-primary endpoints, there were no significant differences between the Active and Control groups in either trial as the posterior ORs were close to 1 and had wide CrIs. In trial 1, the Active group had a lower prevalence of intrusive thoughts (OR 0.82, 95% CrI: 0.48–1.40) and less severe intrusions (OR 0.71, 95% CrI 0.43–1.20) than the Control group. The probability of posterior OR > 1 was 23% for prevalence and 9% for severity (Fig. 3). In trial 2, the Active group had a higher prevalence of intrusive thoughts (OR 1.20, 95% CrI 0.76–1.80) and less severe intrusions (OR 0.95, 95% CrI 0.63–1.40) than the Control group. The probability of posterior OR > 1 was 78% for prevalence and 40% for severity.

For the three secondary endpoints of depressed mood, quality of life and periods of anxiety or worry, there was a tendency with higher impairment among patients in the Active group. In trial 1, the ORs were 1.60 (95% CrI, 0.68–3.40), 1.90 (95% CrI 1.20–3.0) and 1.50 (95% CrI 0.82–2.70) for the three secondary endpoints, respectively. In trial 2, the corresponding ORs were 1.80 (95% CrI 0.78–4.0), 1.60 (95% CrI 1.0–2.40) and 2.60 (95% CrI 1.50–4.30) (Additional file 1, Figure S1.1-S1.3).

The pattern of missing data is raised no concerns regarding the validity of the results (Additional file 1, Figure S3.1-S3.2). The convergence diagnostics indicated that the posterior distribution was adequately estimated (Additional file 1, Figure S4.1-S4.5). The distribution of the observed responses is presented in Additional file 1, Figure S5.1-S5.5. The sensitivity analyses (unadjusted, frequentist and prevalent user analyses) gave similar results to the main analysis (Additional file 1, Figure S6.1-S6.2).

## Discussion

This study combined data from three Swedish longitudinal cohort studies and the Swedish Prescribed Drug Register to examine whether beta-blocker therapy could reduce intrusive thoughts, anxiety, depressed mood, and low quality of life in colorectal or prostate cancer survivors. We emulated a target trial of patients being randomized to treatment with beta-blockers or no treatment with beta-blockers and used a Bayesian analysis to enable a probabilistic interpretation of the estimated causal effects. Based on the analysis results, there was no evidence suggesting that beta-blockers improve the well-being of cancer survivors. Thus, the previous findings of a possible protective effect of beta-blockers on intrusive thoughts in cancer patients could not be verified. The results also indicated that patients who initiated beta-blockers had a tendency of higher impairment with lower quality of life and more frequent periods of anxiety and worry.

One possible reason for higher impairment among patients who initiated beta-blockers could be that they were sensitized by their cancer diagnosis, and thus, an additional diagnosis of cardiovascular disease requiring treatment could cause more distress than otherwise expected. However, our sensitivity analysis produced consistent results, indicating that prevalent users, patients who used beta-blockers before their cancer diagnosis, exhibited higher impairments compared to those who were not using beta-blockers. Another explanation is that beta-blockers are common agents associated with drug-induced nightmares [24], which could affect distress. Contrary to previous reports of reduced intrusive thoughts, in which all patients were women [11], the majority of the patients in this study were men. These sex differences could have influenced the findings [25].

One also must consider that patients with cancer could have intrusive thoughts not just about past events but about future uncertainties. The presumed effect of beta-blockers in patients with PSTD is that they sever the bodily fear response that the traumatic event gave rise to from the memory of the event [9, 26]. As such, when beta-blockers are given soon after the initial traumatic event, or reactivation of the memory of the traumatic event, the neurological association between the patient’s memory and the subsequent emotional response is hindered. Therefore, later recollection of the traumatic event will not give rise to the previously experienced emotional distress. However, as patients with cancer do not necessarily have intrusive thoughts of a specific past event but about future uncertainties, they may not be the best candidates for the protective effects of beta-blockers concerning traumatic experiences. At present, there is insufficient evidence to implement beta-blockers as a prophylactic drug against intrusive thoughts in connection to cancer.

A strength of the study is the combination of registry data with clinical studies for the emulation of a randomized controlled trial. The use of three large multicenter cohort studies of common types of cancers give a good base for generalisation. Data on beta-blocker use were collected from the Swedish Prescribed Drug Register, in which all prescription drugs sold in the country must be registered. The registry distinguishes between prescription and dispensation. While there is uncertainty about adherence to a dispensed drug, studies indicate that adherence is high to prescription drugs [27].

The emulation approach with a clearly defined starting point with baseline values and an incident exposure approach to group assignment, enabled us to limit selection bias [12]. Nevertheless, the analysis cannot fully mimic a randomized controlled trial but rather a pragmatic open-label study. Non-randomized allocation means that we cannot separate the possible effects of cardiovascular disease from those of beta-blockers.

Another weakness is the data sparsity with few patients being assigned to the Active group initiating beta-blockers. The long grace period (time from initiation to actual dispense), that spanned the entire follow-up period, was necessary to allow sufficient numbers of patients to be assigned to the Active group, i.e., initiation of beta-blocker treatment [28]. As beta-blocker works instantly [9] this is not expected to be a factor that would mediate the effect. Due to data sparsity, we had to refrain from including more adjustment variables in the regression models despite the use of regularizing priors. We identified age, alcohol consumption and mental health status at baseline as confounders, however, as we cannot rule out residual confounding, there remains an inherent risk of bias.

## Conclusion

This emulated trial could not find evidence of a protective effect of beta-blockers on intrusive thoughts in colorectal and prostate cancer survivors. Rather, patients who initiated treatment with beta-blockers had lower quality of life and more anxiety than those who did not initiate beta-blockers. This study does not rule out a possible protective effect of beta-blockers on intrusive thoughts, but interventional trials are required to assert the effect of beta-blockers without the confounder of cardiovascular (or other) disease the prescribed beta-blocker was intended as a treatment for.

### Supplementary Information

**Supplementary Material 1.**

## Data Availability

The dataset analyzed during the current study are available from the corresponding author upon reasonable request. The data are not publicly available due to privacy and ethical restrictions.

## Abbreviations

ATC: Anatomic Therapeutic Chemical classification
CrI: Credible intervals
IQR: Interquartile rate
LAPPRO: Laparoscopic Prostatectomy Robot Open
MCMC: Markov Chain Monte Carlo
OR: Odds ratio
PSTD: Post-traumatic stress disorder
QoLiCOL: Quality of Life in Colon Cancer
QoLiRECT: Quality of Life in Rectal Cancer
SAP: Statistical Analysis Plan
