# Supplementary material for: Do beta-blockers reduce negative intrusive thoughts and anxiety in cancer survivors? – An emulated trial
Source: BMC Cancer. 2024 Apr 11;24:447. doi: 10.1186/s12885-024-12236-3 (PMC11007941; doi:10.1186/s12885-024-12236-3)

Supplementary material

Contents

Supplement 1: Additional Tables and Figures 1
Supplement 2: Additional details on the statistical analyses and programming code........ 6
Supplement 3: Characterization of missing data 7
Supplement 4: Convergence diagnostics of the MCMC samplers........ 9
Supplement 5: The distribution of the observed responses........ 14
Supplement 6: Sensitivity analyses........ 19

Supplement 1: Additional Tables and Figures

Table S1.1. Summary of target and emulated trials

**Emulation**		**Summary of protocol of hypothetical target trial**	**Analysis of data**
1(2)	Eligibility criteria	Patients diagnosticed with prostate, colon or rectal cancer scheduled for surgery with curative intent and no use of beta-block therapy in the last 12 months before baseline	Patients scheduled for surgery with no pick up prescription of beta-block therapy in the period 12 months before the preoperative (12 month) questionnaire and returned both questionnaires to the study secretariat
	Treatment strategies	1. Initiate betablock therapy at baseline and remain until follow-up<br>2.Refrain from taking betablock therapy at baseline and remain until follow-up	1. Pick up at least one prescription of beta-block therapy between preoperative (12 month) and 12 month (24 month) questionnaire<br>2. Pick up no prescription of beta-block therapy between preoperative (12 month) and 12 month (24 month) questionnaire.
	Assignment procedures	Participants will be randomly assigned to either strategy at baseline, and will be aware of the strategy they have been assigned to.	Participants will be assigned to respective group according to the definition of treatment strategy.
	Follow-up period	The time point where the patient complete the 12 month questionnaire	The time point where the patient complete the 12 (24) month questionnaire
	Outcome	See Table 1	See Table 1
	Causal contrasts	Intention to treat effect	Intention to treat effect of pick up prescription
	Analysis plan	Intention to treat analysis	Intention to treat effect of pick up prescription with handling of confounders by by regression adjustment and preoperative outcome as covariate (ANCOVA)

Table S1.2. Study participant characteristics

	Trial 1			Trial 2		
Characteristic	Overall, N = 4,020[1]	Control, N = 3,961	Active, N = 59	Overall, N = 3,219[1]	Control, N = 3,132	Active, N = 87
**Source, n / N (%)**						
LAPPRO	2,582 / 4,020 (64%)	2,552 / 3,961 (64%)	30 / 59 (51%)	2,771 / 3,219 (86%)	2,700 / 3,132 (86%)	71 / 87 (82%)
QoliCOL	923 / 4,020 (23%)	903 / 3,961 (23%)	20 / 59 (34%)	0 / 3,219 (0%)	0 / 3,132 (0%)	0 / 87 (0%)
QoliRECT	515 / 4,020 (13%)	506 / 3,961 (13%)	9 / 59 (15%)	448 / 3,219 (14%)	432 / 3,132 (14%)	16 / 87 (18%)
**Sex, n / N (%)**						
Female	688 / 4,020 (17%)	674 / 3,961 (17%)	14 / 59 (24%)	189 / 3,219 (5.9%)	181 / 3,132 (5.8%)	8 / 87 (9.2%)
Male	3,332 / 4,020 (83%)	3,287 / 3,961 (83%)	45 / 59 (76%)	3,030 / 3,219 (94%)	2,951 / 3,132 (94%)	79 / 87 (91%)
**Age, Median (IQR)**	65 (60, 70)	65 (60, 70)	67 (62, 74)	64 (59, 68)	64 (59, 68)	65 (62, 69)
**BMI, Median (IQR)**	25.7 (23.8, 27.8)	25.7 (23.8, 27.8)	25.6 (24.4, 27.6)	25.7 (23.9, 27.8)	25.7 (23.9, 27.8)	25.6 (24.3, 28.1)
Unknown	349	342	7	404	401	3
**Mental health issues, n / N (%)**						
No	3,402 / 3,717 (92%)	3,358 / 3,662 (92%)	44 / 55 (80%)	2,699 / 3,077 (88%)	2,632 / 2,996 (88%)	67 / 81 (83%)
Yes	315 / 3,717 (8.5%)	304 / 3,662 (8.3%)	11 / 55 (20%)	378 / 3,077 (12%)	364 / 2,996 (12%)	14 / 81 (17%)
Unknown	303	299	4	142	136	6
**Binge drinking, n / N (%)**						
No	3,140 / 3,966 (79%)	3,096 / 3,907 (79%)	44 / 59 (75%)	2,528 / 3,183 (79%)	2,460 / 3,096 (79%)	68 / 87 (78%)
Yes	826 / 3,966 (21%)	811 / 3,907 (21%)	15 / 59 (25%)	655 / 3,183 (21%)	636 / 3,096 (21%)	19 / 87 (22%)
Unknown	54	54	0	36	36	0
**Intrusive thoughts, prevalence, n / N (%)**						
Never	970 / 3,745 (26%)	956 / 3,689 (26%)	14 / 56 (25%)	1,451 / 3,149 (46%)	1,420 / 3,065 (46%)	31 / 84 (37%)
Less often than once a week	1,281 / 3,745 (34%)	1,266 / 3,689 (34%)	15 / 56 (27%)	1,090 / 3,149 (35%)	1,059 / 3,065 (35%)	31 / 84 (37%)
At least once a week	670 / 3,745 (18%)	659 / 3,689 (18%)	11 / 56 (20%)	374 / 3,149 (12%)	360 / 3,065 (12%)	14 / 84 (17%)
At least three times a week	824 / 3,745 (22%)	808 / 3,689 (22%)	16 / 56 (29%)	234 / 3,149 (7.4%)	226 / 3,065 (7.4%)	8 / 84 (9.5%)
Unknown	275	272	3	70	67	3
**Intrusive thoughts, severity, n / N (%)**						
Never had	810 / 4,004 (20%)	799 / 3,945 (20%)	11 / 59 (19%)	1,183 / 3,211 (37%)	1,157 / 3,124 (37%)	26 / 87 (30%)
Not intrusive at all	1,084 / 4,004 (27%)	1,064 / 3,945 (27%)	20 / 59 (34%)	1,172 / 3,211 (36%)	1,148 / 3,124 (37%)	24 / 87 (28%)
Somewhat intrusive	1,104 / 4,004 (28%)	1,093 / 3,945 (28%)	11 / 59 (19%)	582 / 3,211 (18%)	555 / 3,124 (18%)	27 / 87 (31%)
More than somewhat intrusive	1,006 / 4,004 (25%)	989 / 3,945 (25%)	17 / 59 (29%)	274 / 3,211 (8.5%)	264 / 3,124 (8.5%)	10 / 87 (11%)
Unknown	16	16	0	8	8	0
**Depressed mood, n / N (%)**						
No	3,622 / 3,979 (91%)	3,576 / 3,920 (91%)	46 / 59 (78%)	3,016 / 3,194 (94%)	2,936 / 3,108 (94%)	80 / 86 (93%)
Yes	357 / 3,979 (9.0%)	344 / 3,920 (8.8%)	13 / 59 (22%)	178 / 3,194 (5.6%)	172 / 3,108 (5.5%)	6 / 86 (7.0%)
Unknown	41	41	0	25	24	1
**Quality of life, n / N (%)**						
High	579 / 3,980 (15%)	570 / 3,921 (15%)	9 / 59 (15%)	617 / 3,203 (19%)	602 / 3,116 (19%)	15 / 87 (17%)
Low	1,248 / 3,980 (31%)	1,230 / 3,921 (31%)	18 / 59 (31%)	1,196 / 3,203 (37%)	1,174 / 3,116 (38%)	22 / 87 (25%)
Intermediate	1,099 / 3,980 (28%)	1,089 / 3,921 (28%)	10 / 59 (17%)	873 / 3,203 (27%)	843 / 3,116 (27%)	30 / 87 (34%)
Very low	1,054 / 3,980 (26%)	1,032 / 3,921 (26%)	22 / 59 (37%)	517 / 3,203 (16%)	497 / 3,116 (16%)	20 / 87 (23%)
Unknown	40	40	0	16	16	0
**Periods of intense anxiety, worry or panic, n / N (%)**						
Never	2,451 / 3,989 (61%)	2,424 / 3,930 (62%)	27 / 59 (46%)	2,678 / 3,211 (83%)	2,613 / 3,125 (84%)	65 / 86 (76%)
Less often than once a week	431 / 3,989 (11%)	418 / 3,930 (11%)	13 / 59 (22%)	261 / 3,211 (8.1%)	250 / 3,125 (8.0%)	11 / 86 (13%)
At least once a week	1,107 / 3,989 (28%)	1,088 / 3,930 (28%)	19 / 59 (32%)	272 / 3,211 (8.5%)	262 / 3,125 (8.4%)	10 / 86 (12%)
Unknown	31	31	0	8	7	1

[1]n / N (%); Median (IQR)

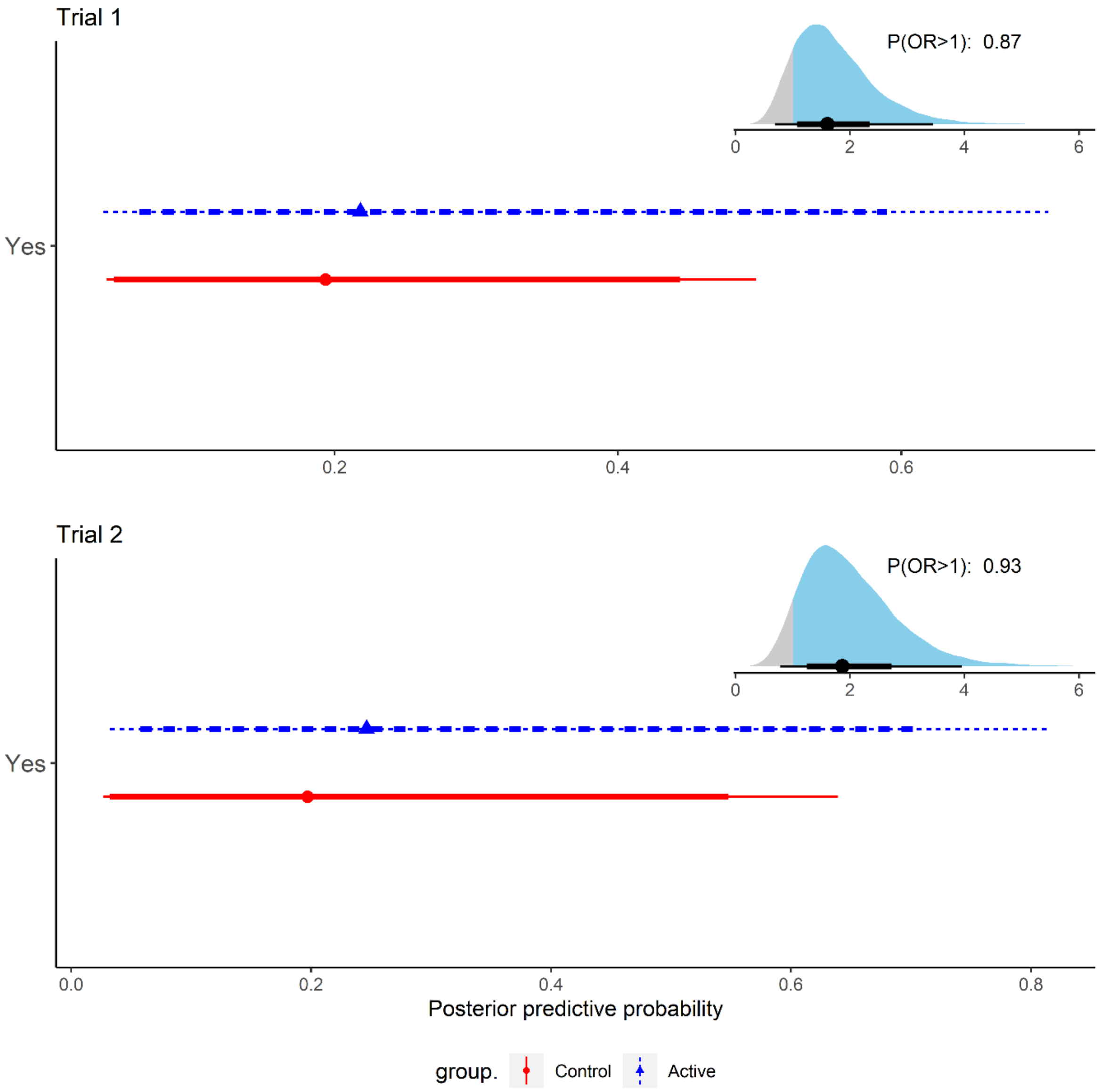

Figure S1.1. Self-reported as depressed. Posterior predictive distribution for the response categories and posterior distribution for the OR.

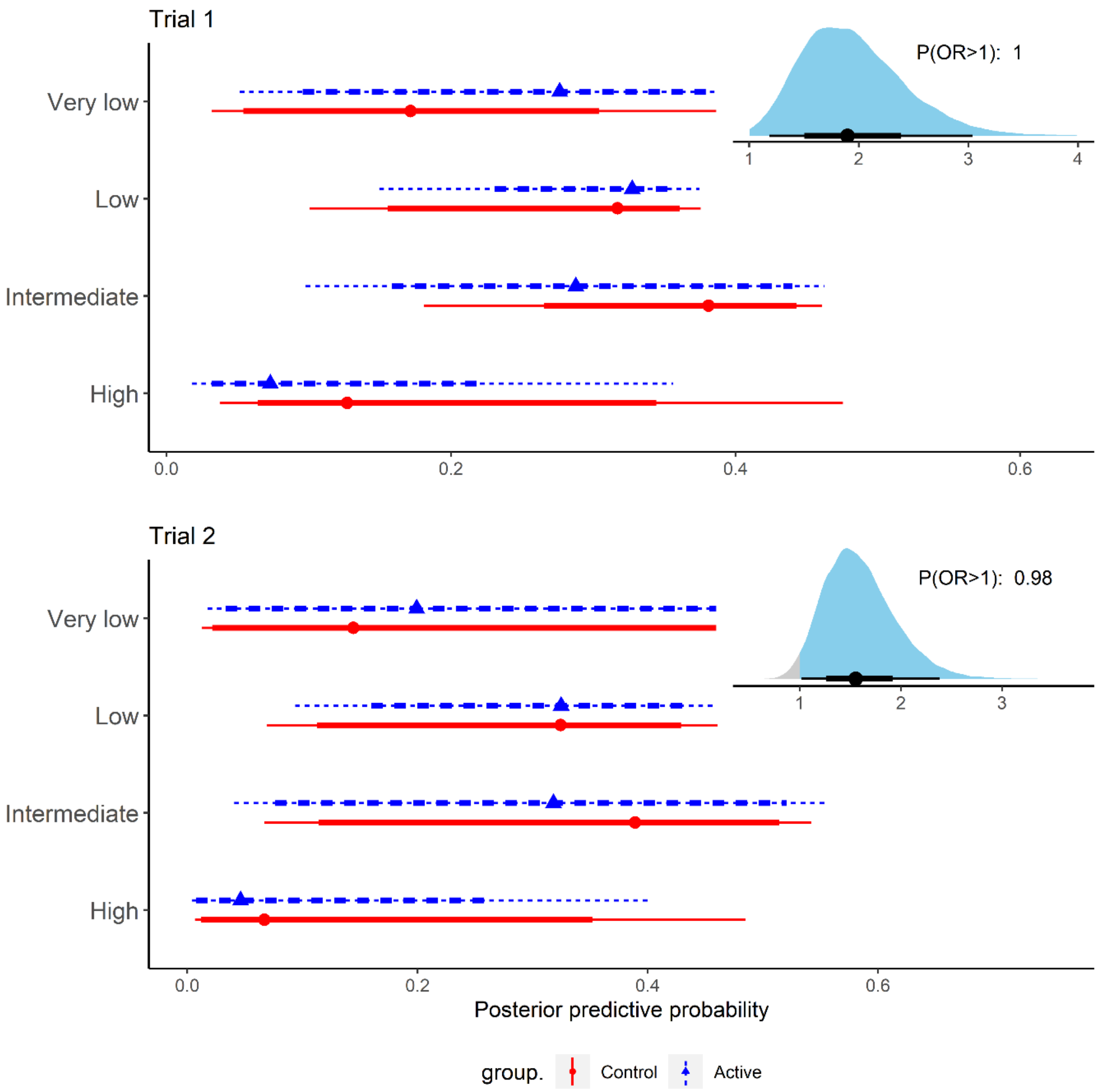

Figure S1.2. Quality of life. Posterior predictive distribution for the response categories and posterior distribution for the OR.

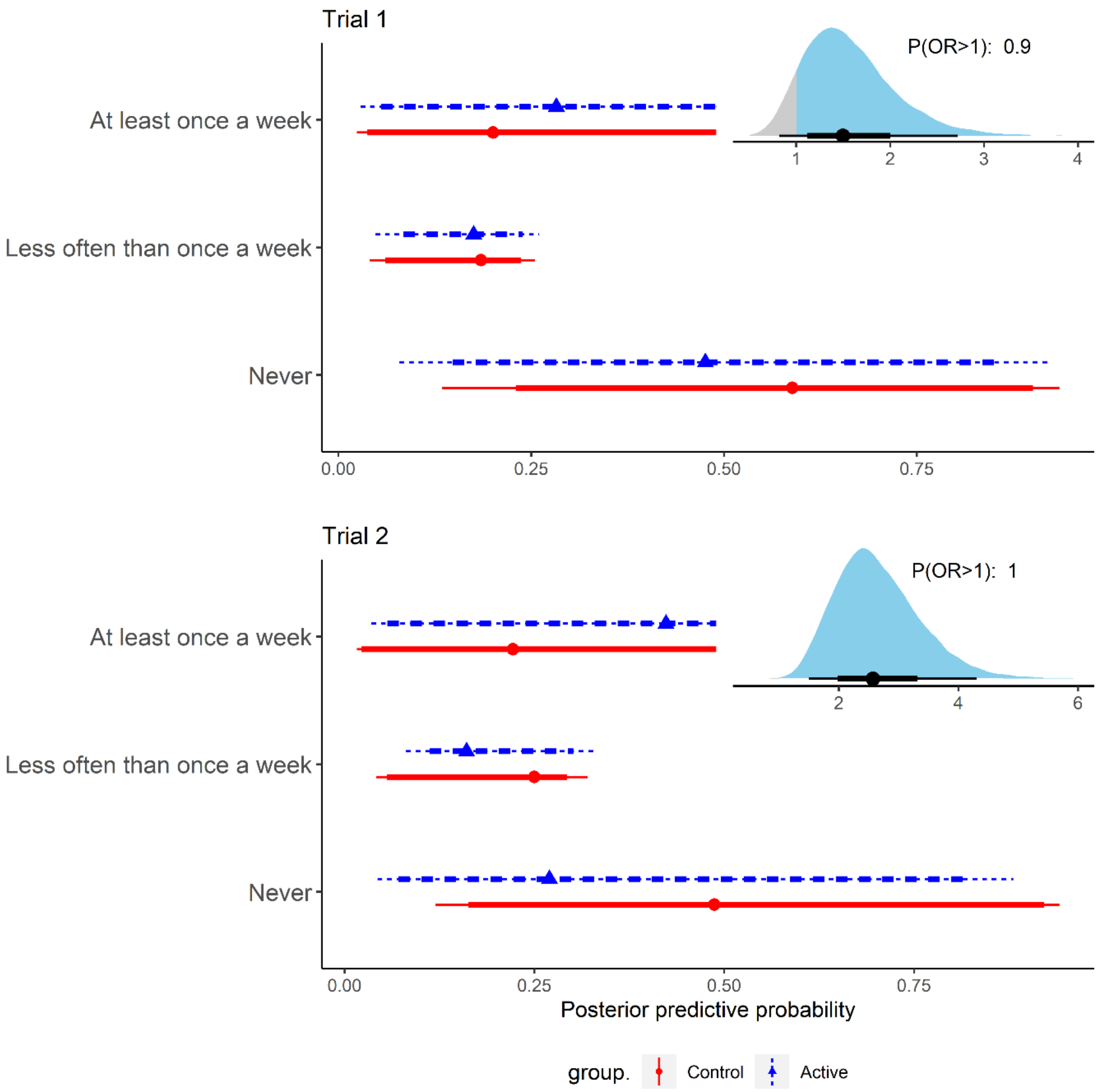

Figure S1.3. Periods of intense anxiety, worry or panic. Posterior predictive distribution for the response categories and posterior distribution for the OR.

Supplement 2: Additional details on the statistical analyses and programming code

Statistical models

Let Y_i be the response for subject $i, i = 1,2,...N$ att follow-up where Y_i takes values in the ordered categories $1,2,..,J$ and is characterized as

$$P(Y_i \leq y) = g\left(\theta_j + \delta \cdot Group + \phi \cdot y_0 + x'\beta\right)$$

$$\theta_j, \delta, \beta \sim Normal(0,1)$$

$$\phi \sim Dirichlet(\alpha)$$

where $g(z) = 1/(1 + exp(-z)$, θ_j are intercepts, δ is the log-odds of subjects in the Active group responding with higher categories than subjects in the Control group, ϕ is the log-odds of change in the baseline response Y_0 and β is the vector of corresponding parameters for the covariates age, alcohol consumption and signs of depression/poor mental health and α is a vector of length J consisting parameter values equal to 2.

Code for main analyses

```
library(brms)
prior. = c(prior(normal(0, 1), class=Intercept),
           prior(normal(0, 1), class=b),
           prior(dirichlet(2, 2, 2), class = simo, coef = Y0) )

brm_multiple( family = cumulative,
              bf(Y  ~ 1 + group + mo(Y0) +
                     age + Alcohol.consumption + Mental.health,
                decomp = "QR"),
              prior = prior., iter=3000, thin = 2,
              seed=777,
              control = list(adapt_delta = 0.95), backend = "cmdstanr",
              cores = 4, chains = 4, normalize = FALSE)
```

Code for the frequentist sensitivity analyses

```
library(MASS)
library(mice)

out <- with(polr(Y  ~ 1 + group + Y0 +
                   age + Alcohol.consumption + Mental.health,
                   Hess = TRUE))
pool  <- as.data.frame(tidy(pool(out) , conf.int = T,  conf.level = 0.95))
```

<u>**Illustrative sample size calculation**</u>

For illustration, assuming a prevalence of intrusive thoughts at least once per week of 35% and 22.5% at diagnosis of non-users of beta-blockers and users of beta-blockers, respectively (beta-blockers reduce prevalence by 12.5%, that is an odds ratio of 0.54 for Active vs Control). Then if there are 40 non-users on each user (1:40), there will be 80% power to detect the 12.5% reduction with a total of 3708 (3619 and 90, respectively) evaluable patients using a two-sided test at 5% significance level.

Supplement 3: Characterization of missing data

The plots are showing in black the location of missing values across the participants (horizontal axis, label suppressed) and across the different variables (vertical axis) and providing information on the total percentage of missing values for each variable

Figure S3.1. Trial 1.

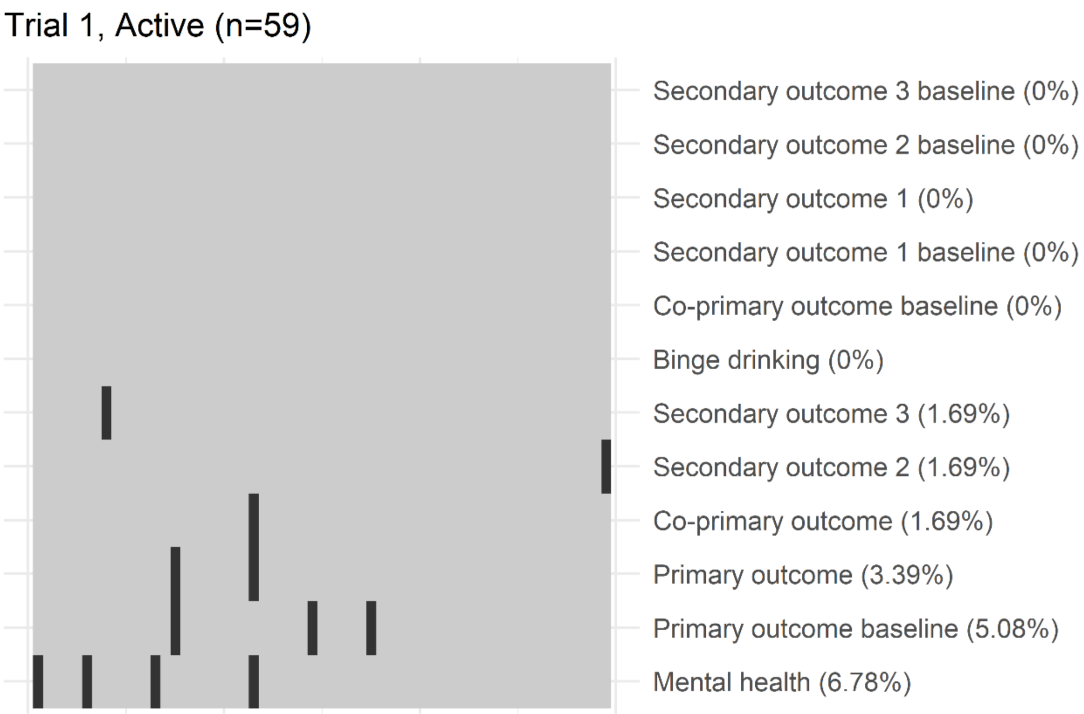

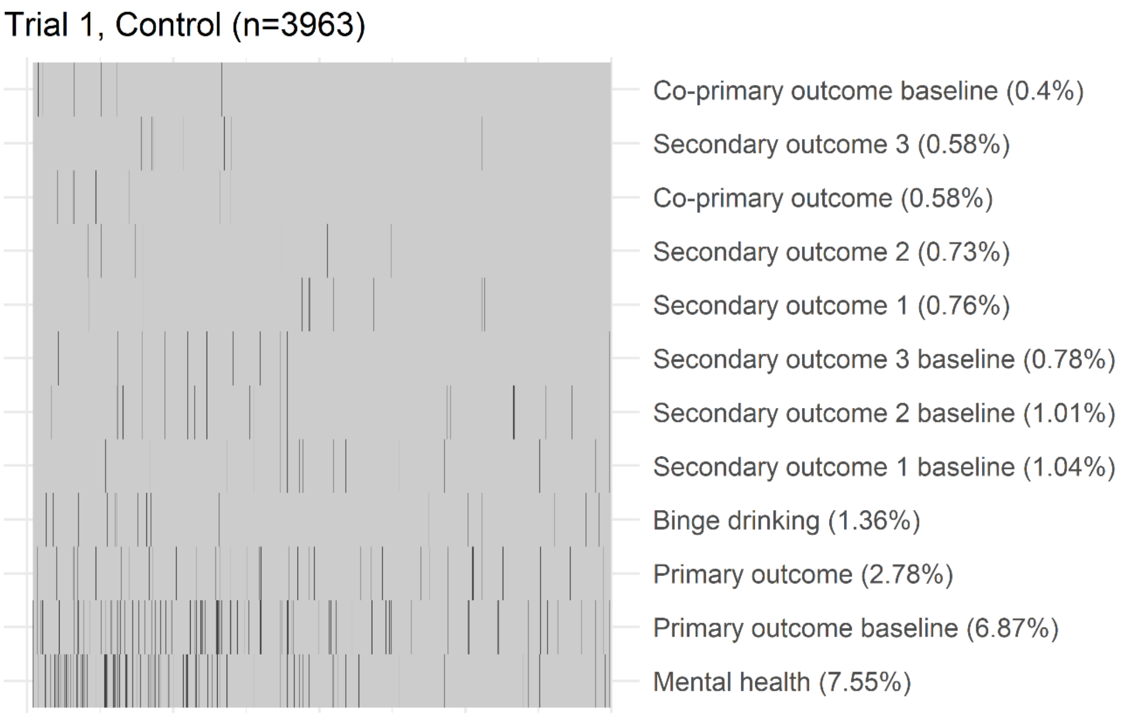

Figure S3.2. Trial 2.

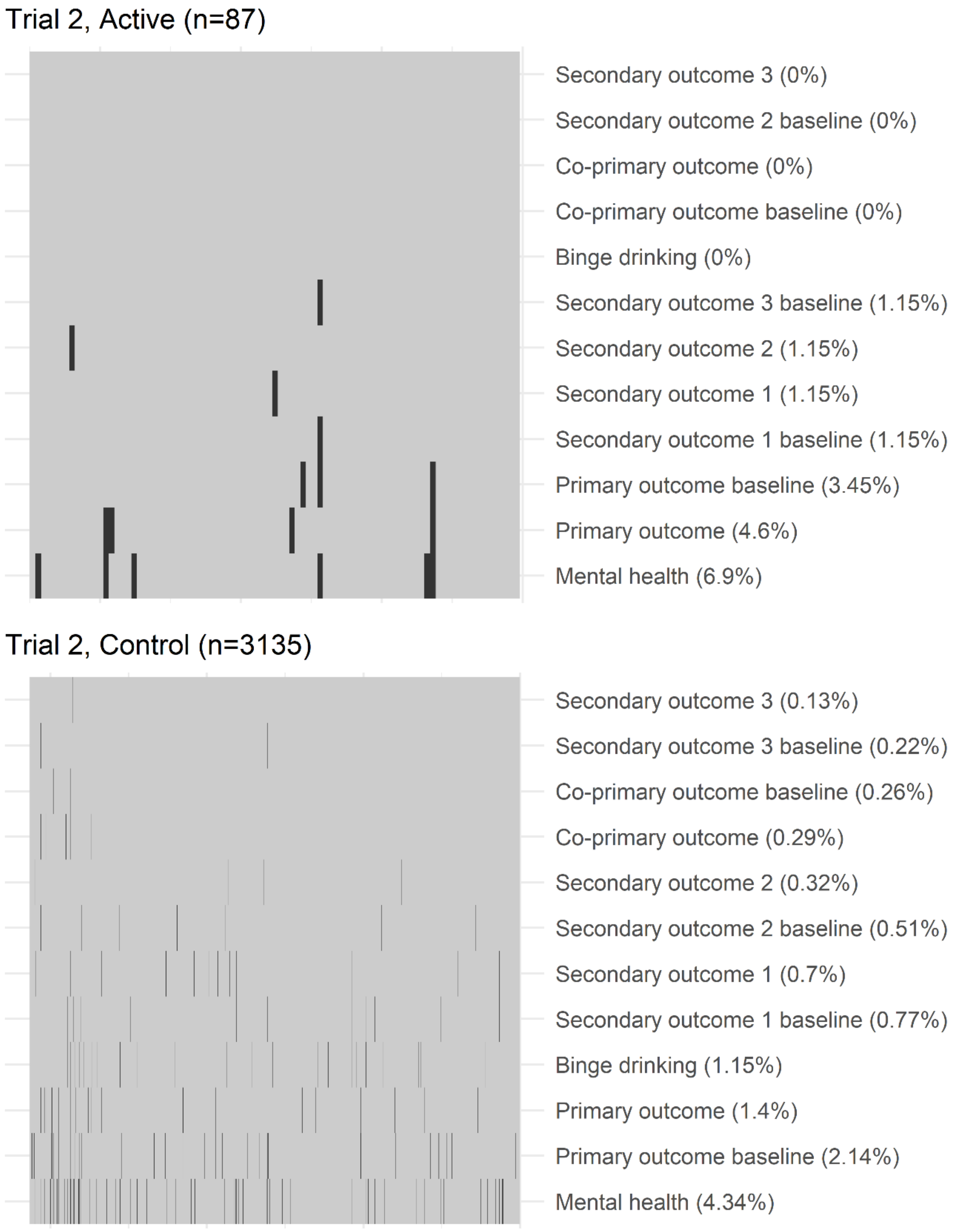

Supplement 4: Convergence diagnostics of the MCMC samplers

The plots are showing the posterior densities for each chain (four) across the five imputed data sets (a total of 20 chains). The differences across chains are primarily due to differences between the imputed data sets.

R-hat is a convergence diagnostic, which compares the between- and within-chain estimates for the parameters. If the chains have not mixed well that is there are discrepancies between the posteriors for the different chains, R-hat is larger than 1.

R-hat is presented by median(min; max) across imputations.

All chains converged well as suggested by R-hat.

Figure S4.1. Primary outcome

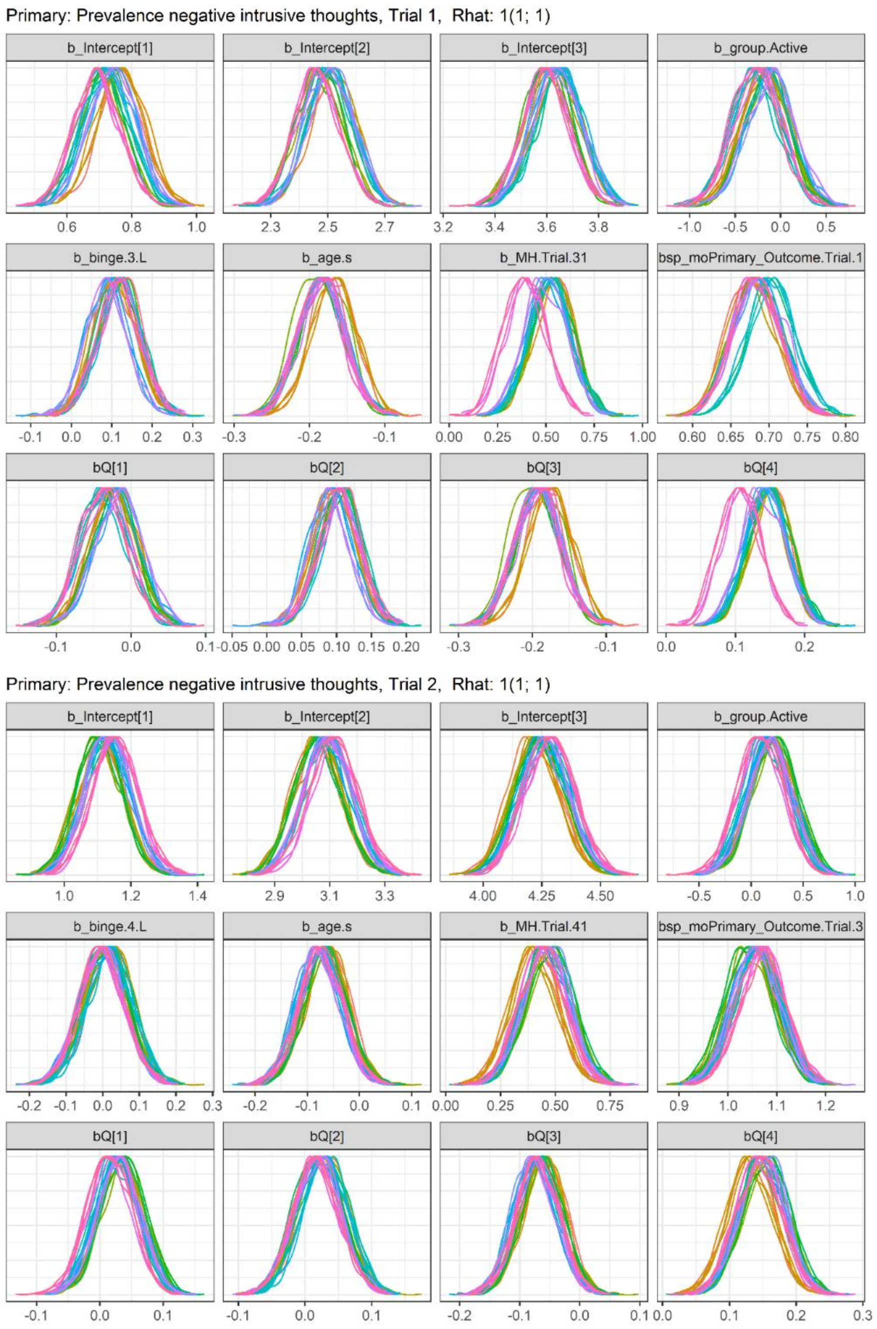

Figure S4.2. Co-primary outcome

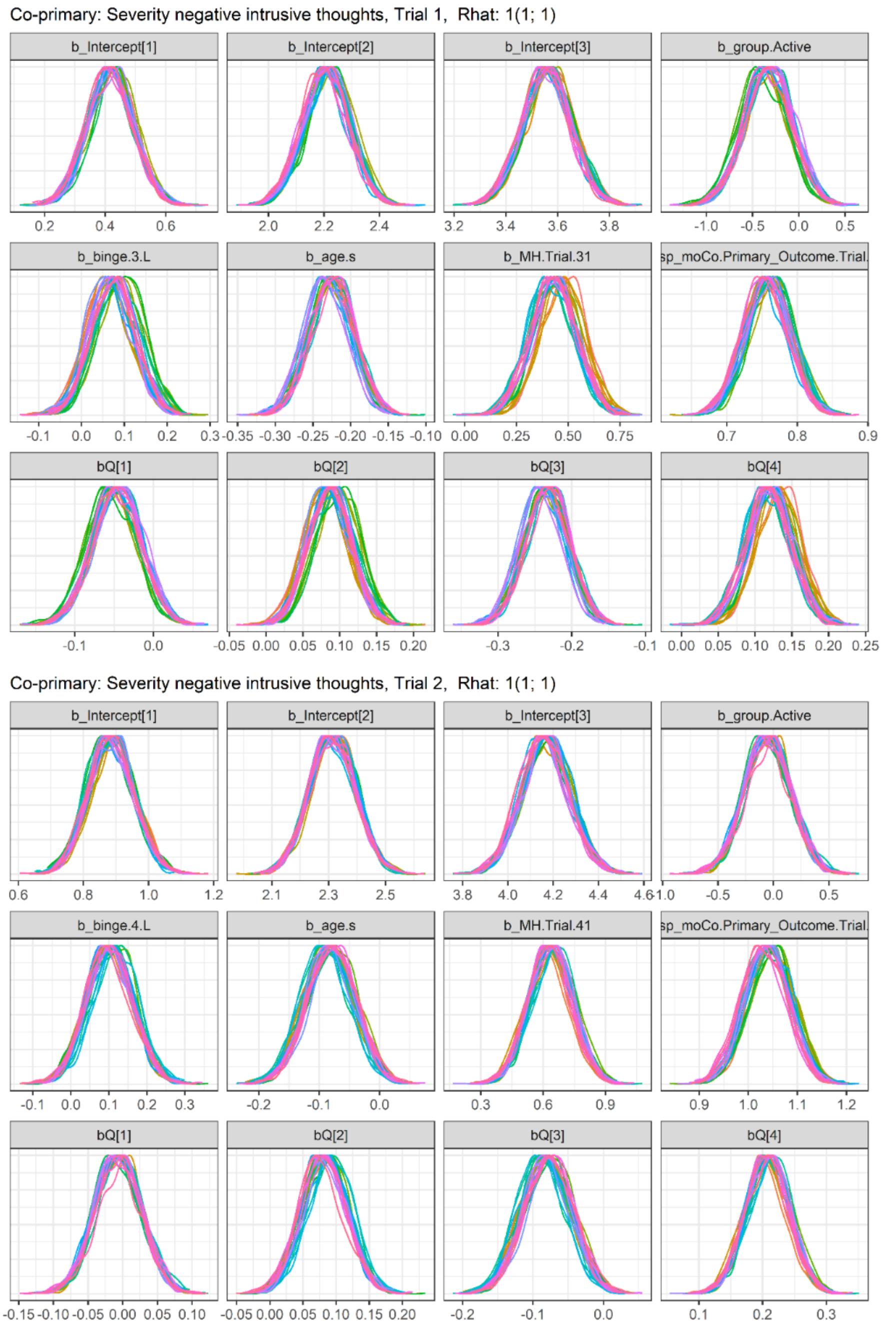

Figure S4.3. Secondary 1 outcome

Secondary 1: Would you call yourself depressed?, Trial 1, Rhat: 1(1; 1)

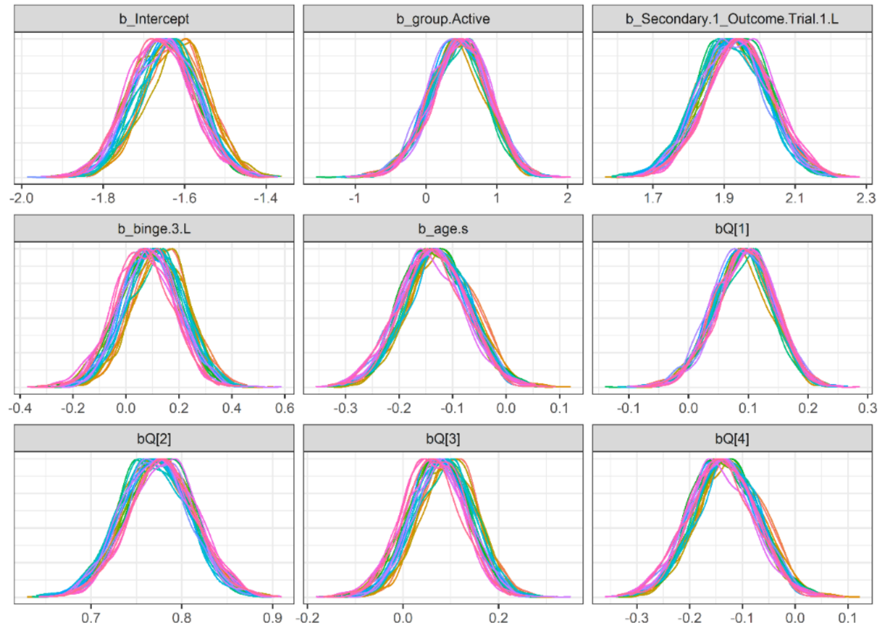

Secondary 1: Would you call yourself depressed?, Trial 2, Rhat: 1(1; 1)

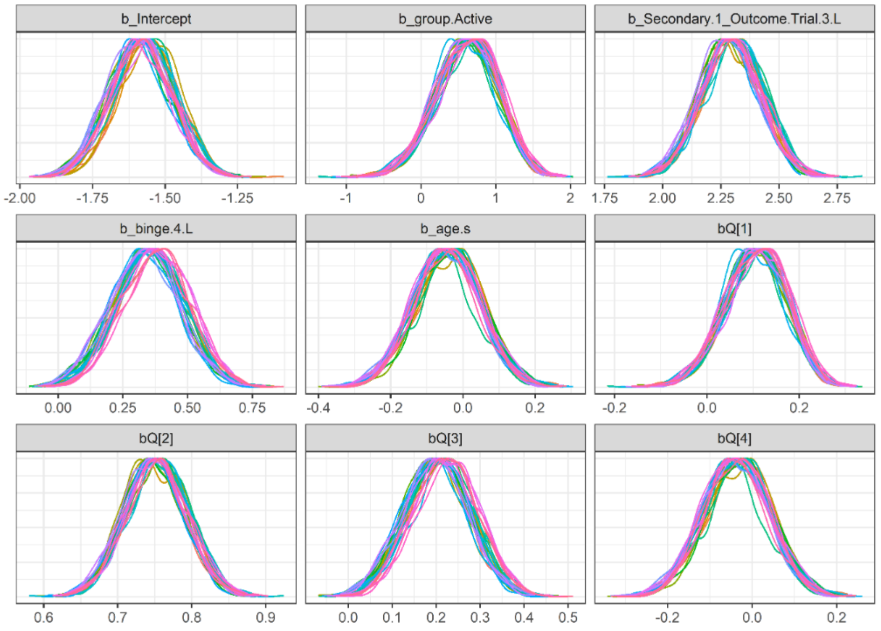

Figure S4.4. Secondary 2 outcome

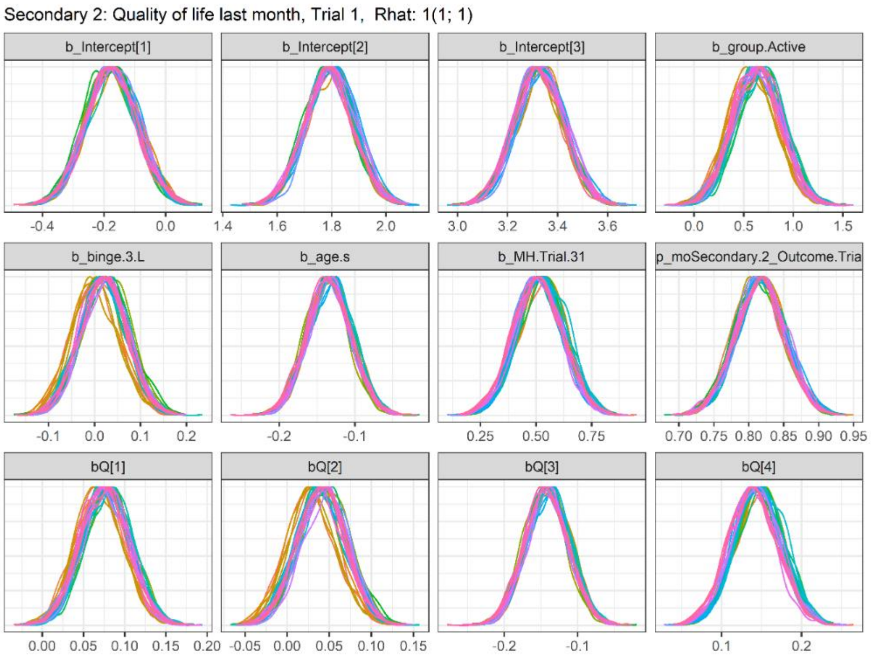

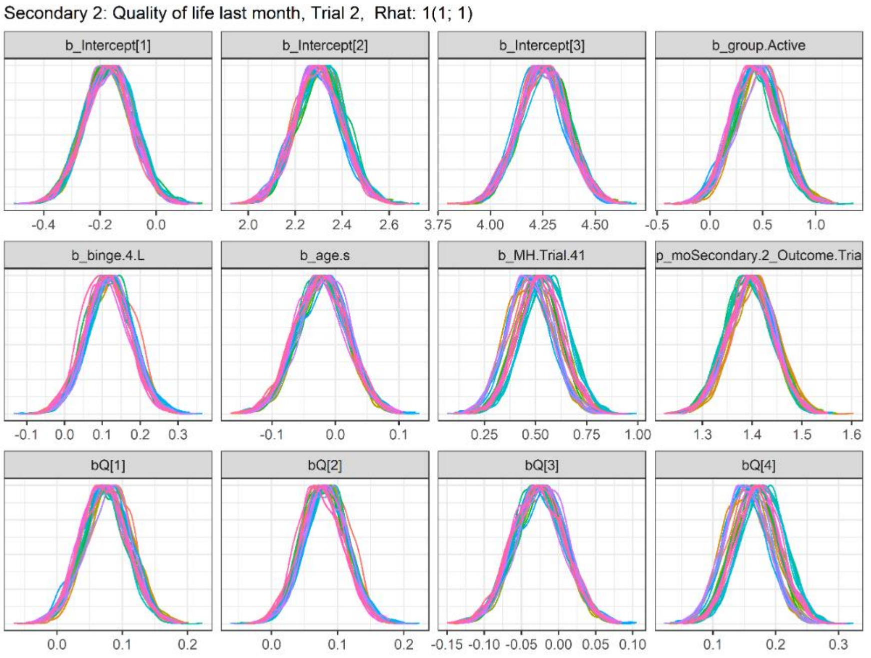

Figure S4.5. Secondary 3 outcome

Secondary 3: Periods of intense anxiety, worry or panic, Trial 1, Rhat: 1(1; 1)

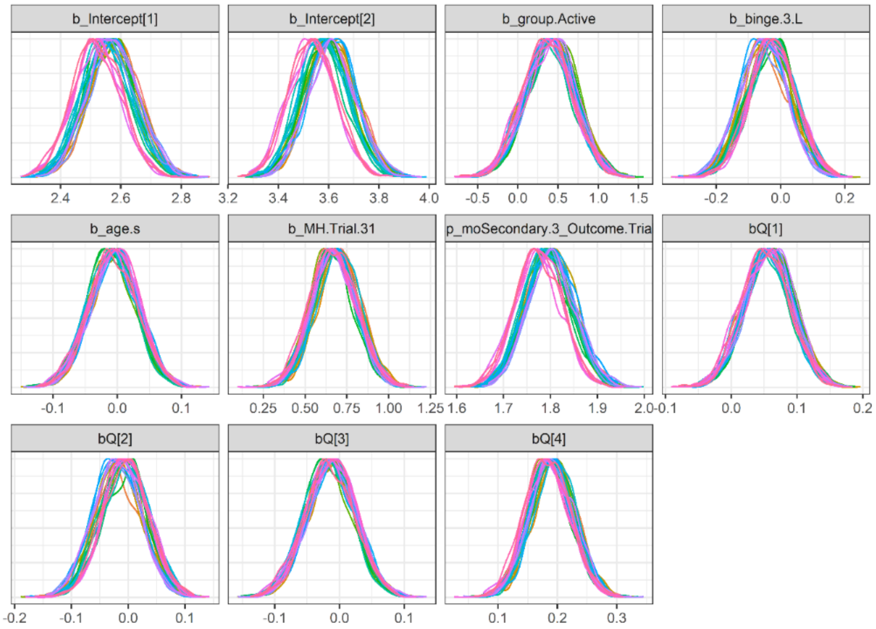

Secondary 3: Periods of intense anxiety, worry or panic, Trial 2, Rhat: 1(1; 1)

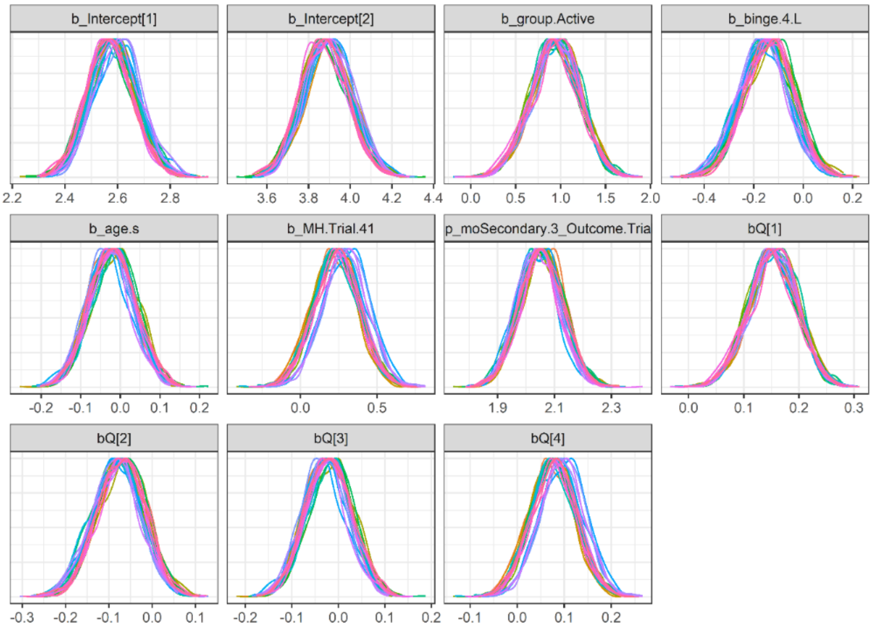

Supplement 5: The distribution of the observed responses

Figured S5.1. Primary outcome

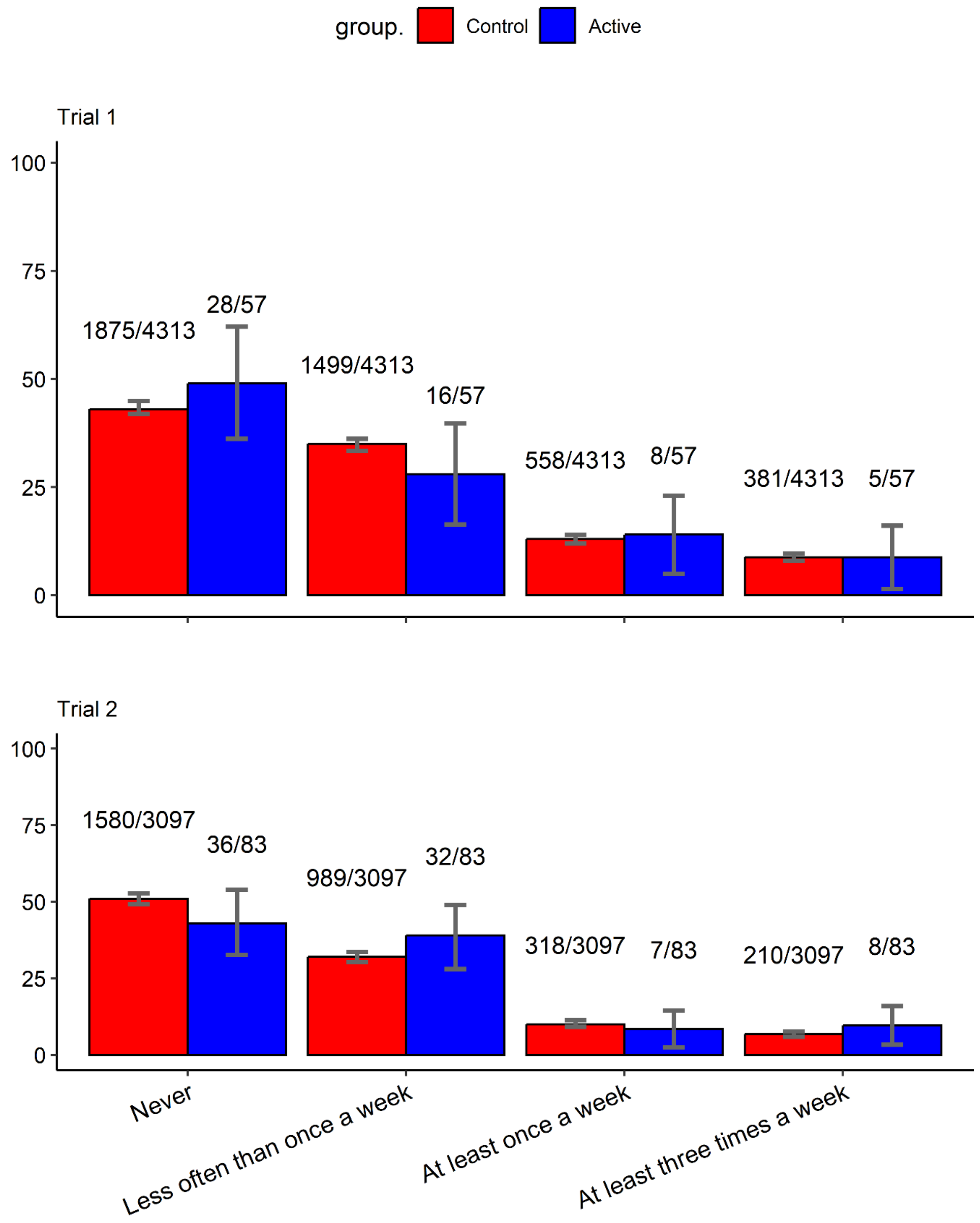

Figure S5.2. Co-primary outcome

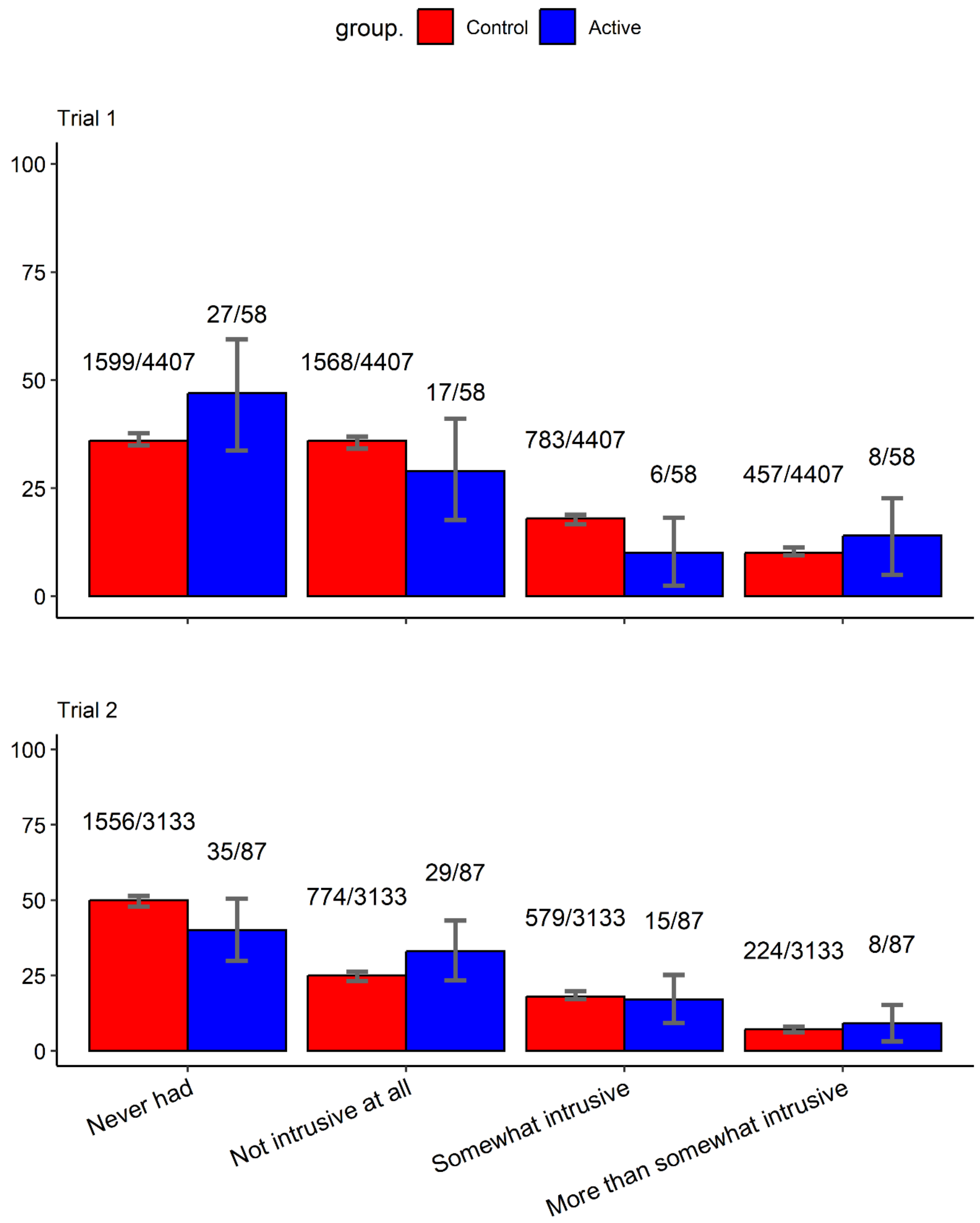

Figure S5.3. Secondary outcome 1

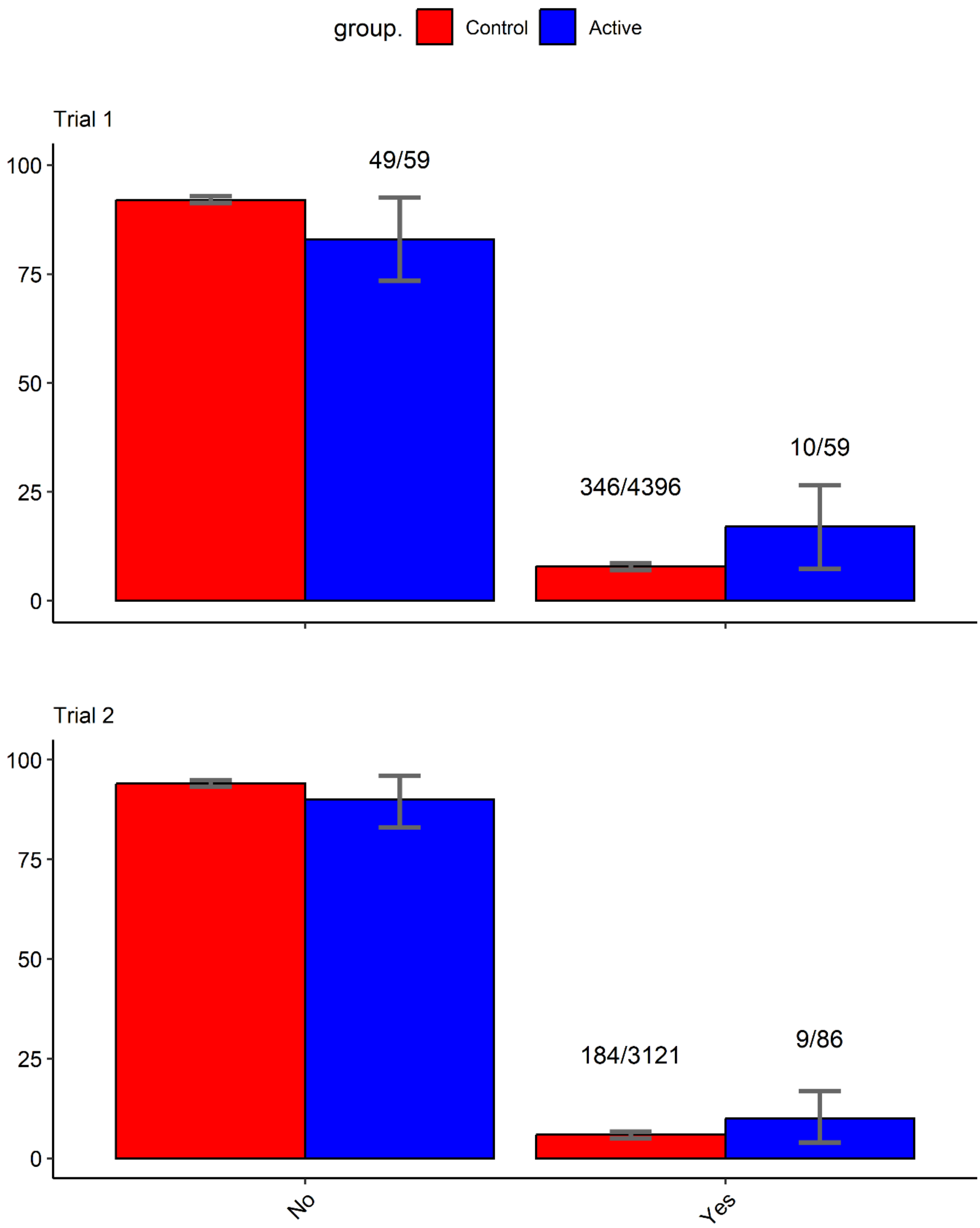

Figure S5.4. Secondary outcome 2

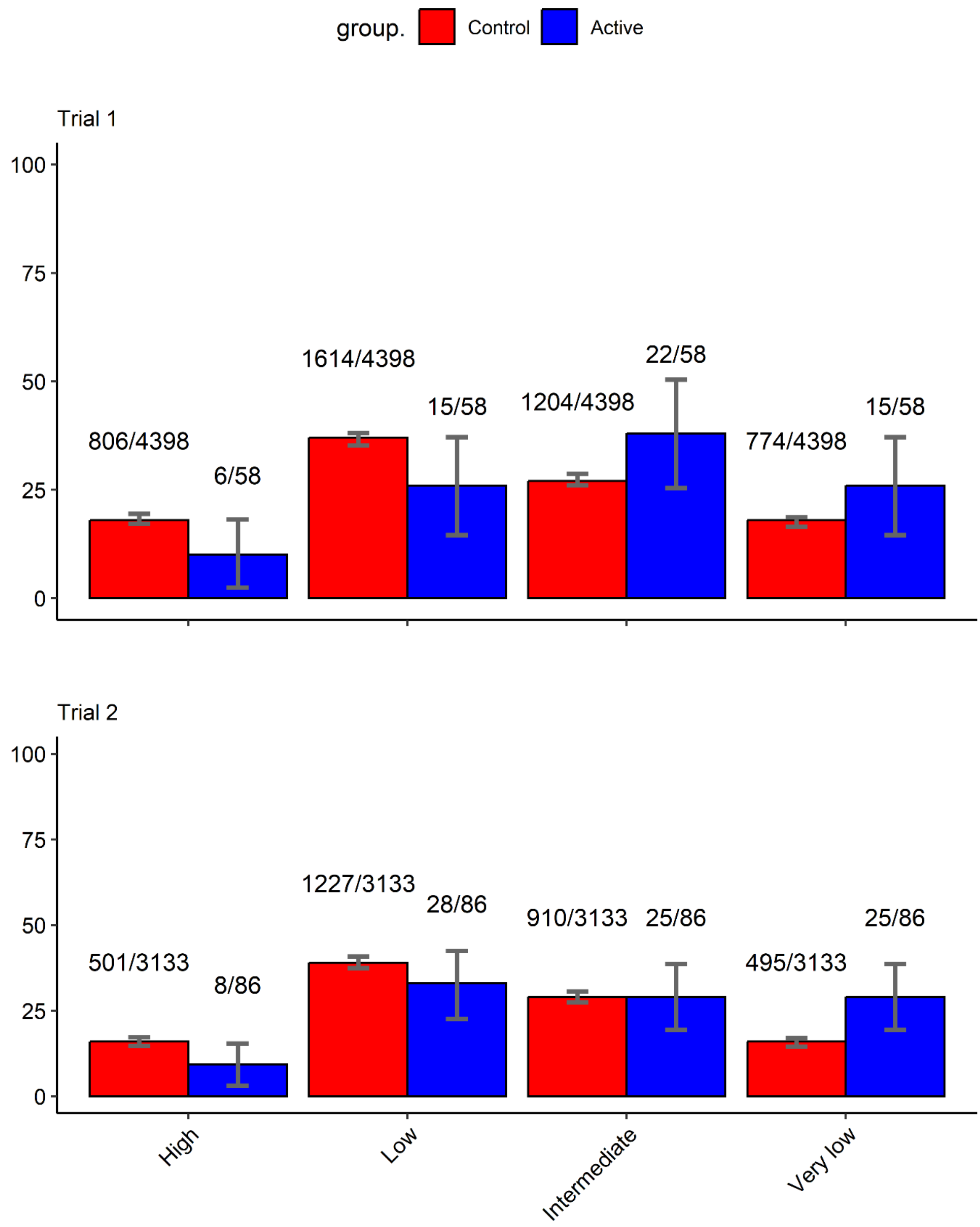

Figure S5.5. Secondary outcome 3

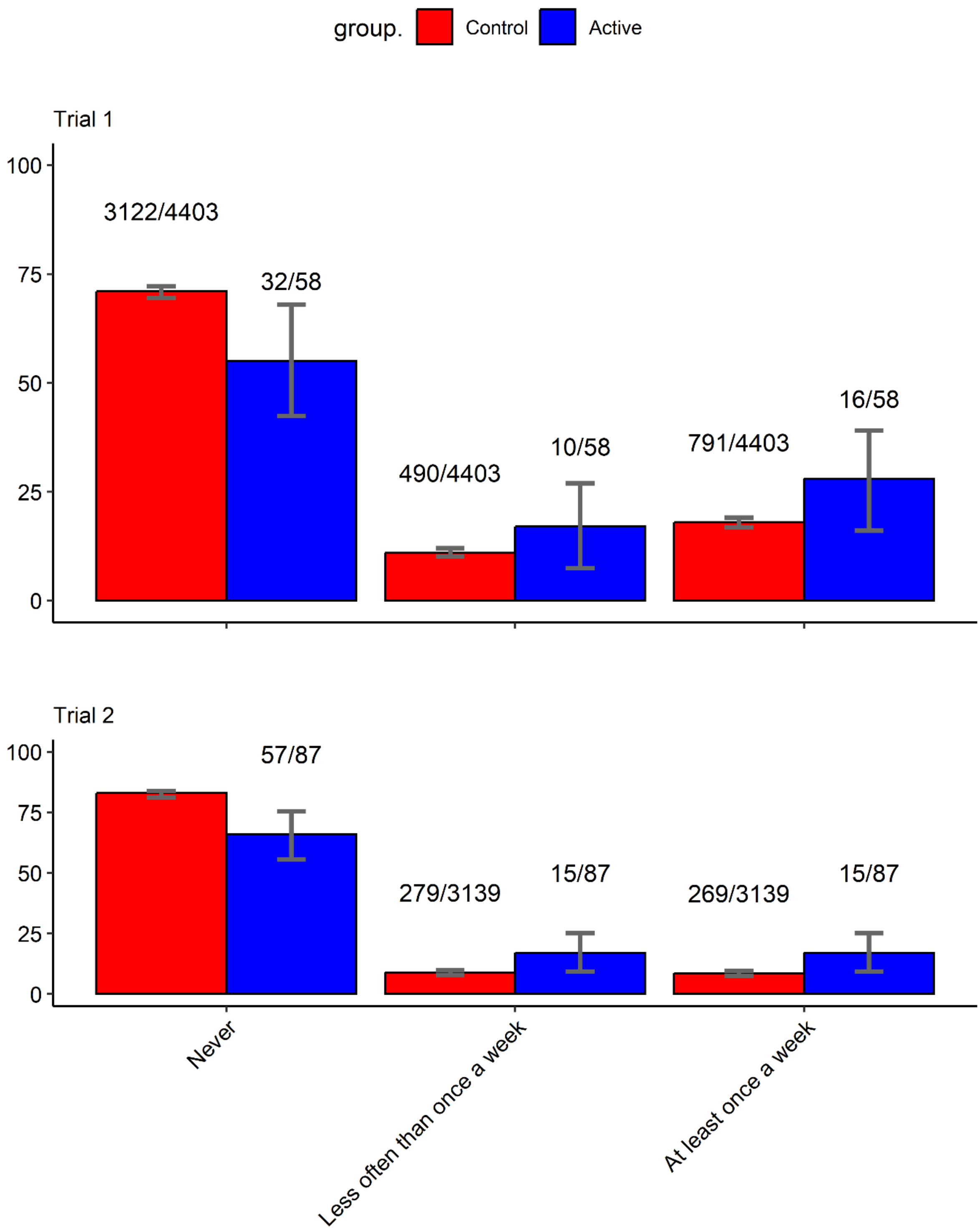

Supplement 6: Sensitivity analyses

Results from the unadjusted complete case (Unadjusted), adjusted frequentist (Frequentist) as well as the main analyses (Main) are presented in the plot below.

Figure S6.1. Results of the sensitivity and the main analyses

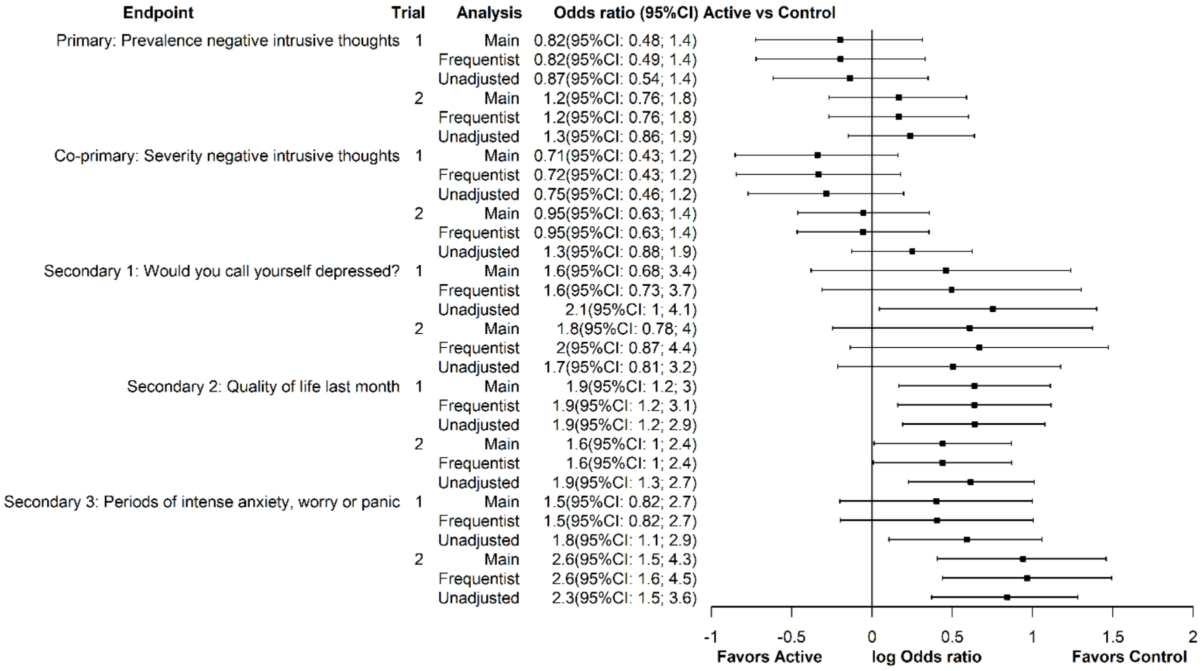

Figure S6.2. Results of the sensitivity analyses of prevalent users.

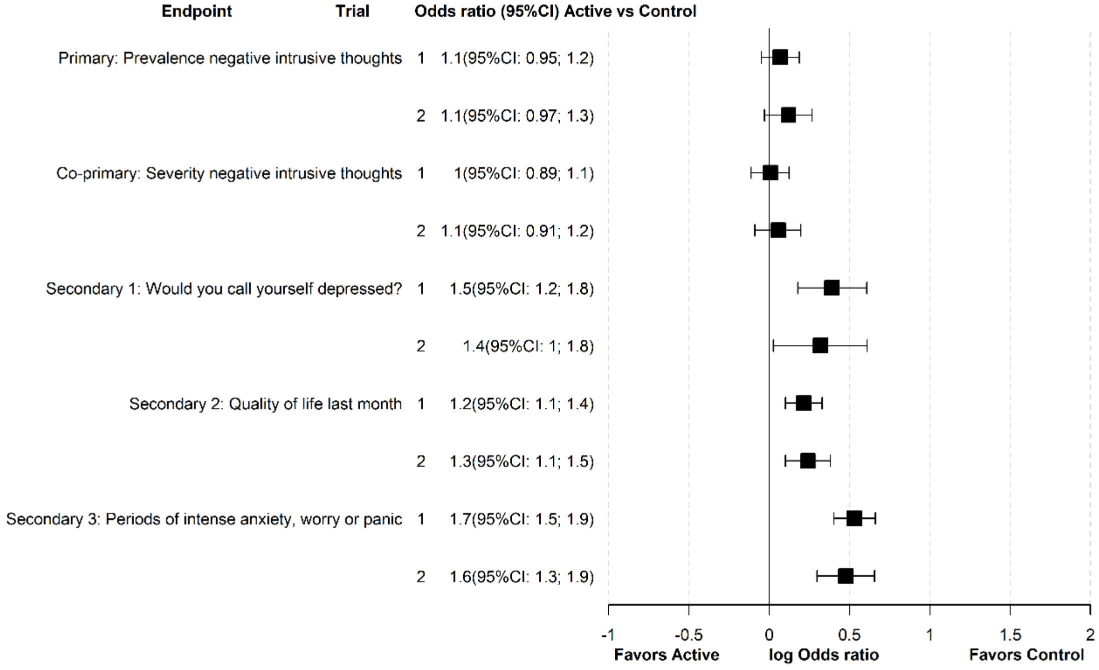

Supplement: Supplementary file 1 — Supplementary Material 1. [file 12885_2024_12236_MOESM1_ESM.pdf]
